# Malignant Atrophic Papulosis Presenting with Intestinal Perforation: A Case Report

**DOI:** 10.31729/jnma.8192

**Published:** 2023-06-30

**Authors:** Asmita Paudel, Min Raj Bhurtel, Ashok Gautam, Amrit Gautam, Mamata Bista, Pragyat Singh

**Affiliations:** 1 Department of Internal Medicine, Pokhara Academy of Health Sciences, Ramghat, Pokhara, Nepal; 2 Department of Internal Medicine, Manipal College of Medical Sciences, Fulbari, Pokhara, Nepal; 3 Department of Infectious Disease, Sukraraj Tropical and Infectious Disease Hospital, Teku, Kathmandu, Nepal; 4 Department of Internal Medicine, Chhatrapati Free Clinic Hospital, Chhatrapati, Kathmandu, Nepal

**Keywords:** *case reports*, *intestinal perforation*, *malignant atrophic papulosis*, *ulcer*, *vasculitis*

## Abstract

Malignant atrophic papulosis sometimes known as Degos' disease is an idiopathic, uncommon condition with fewer than 200 occurrences documented. It is a chronic thrombo-obliterative vasculopathy characterised by papular skin lesions with a core porcelain-white atrophy and a surrounding telangiectatic border. We report a 15-year-old male patient with a recurrent history of hollow viscus perforation, which was managed on all the occasions with exploratory laparotomy and primary perforation repair. Additionally, the patient had a five month history of numerous, non-itchy, atrophic papules with a core porcelain-like area and hyperkeratotic margins, characteristic of Degos' disease. The only basis for diagnosis is the distinctive skin lesions with biopsy. Along with systemic lupus erythematosus and other connective tissue diseases, tuberculosis must also be taken into account while assessing the clinical presentation of malignant atrophic papulosis. There is currently no known treatment for malignant atrophic papulosis that has been effective.

## INTRODUCTION

Malignant atrophic papulosis (MAP) is a rare obliterative vasculopathy,^1^ mostly sporadic in occurrence. The disease first manifests as a skin rash, later progressing to systemic involvement.^2,3^ Malignant atrophic papulosis, is owed by some authors to coagulopathy, vasculitis, or endothelial dysfunction.^4,5^ The prognosis of Degos disease depends on systemic involvement. A malignant form carries a 50% risk of mortality within 2 to 3 years after symptoms appear.^6^ Here, we present a case of intestinal perforation due to Degos disease in a 15-year-old boy.

## CASE REPORT

**Figure 1:**
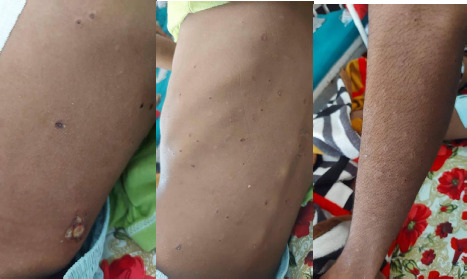
A) First appearance of the lesion on the waist, B) Porcelain white atrophic papules with surrounding erythema present over the back, C) Chest, and limbs.

A 15-year-old boy presented to the Emergency Department of Pokhara Academy of Health Sciences with a history of severe acute abdominal pain for 1 day, initially around the umbilicus, which later generalized all over the abdomen. The pain was associated with fever and non-bilious vomiting. There was a history of skin lesions for five months which first appeared on his waist (Figure 1A) and later on his chest, back (Figure 1B) and limbs (Figure 1C) (Figure 1).

He had no history of joint pain or proximal muscle weakness. There was no history of altered bowel habits. There was no history of tuberculosis (TB) contact either.

**Figure 2:**
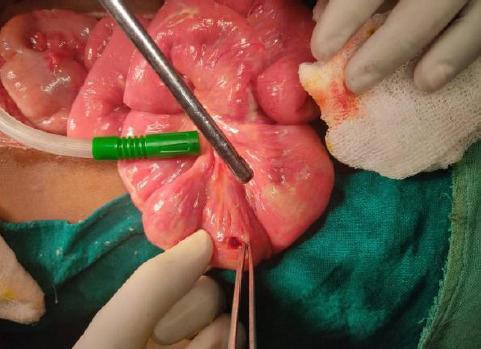
Exploratory laparotomy and primary repair of ileal perforation.

On examination, the abdomen was soft and tender, with rebound tenderness present all over the abdomen. Free gas under the right dome of the diaphragm was seen on the X-ray abdomen. Abdominal ultrasonography (USG) revealed multiple dilated bowel loops. Because of peritonitis secondary to ileal perforation, exploratory laparotomy and primary repair of ileal perforation were done (Figure 2).

Intraoperative findings included two perforated ulcers of approximately 3 mm and 1 mm in size, respectively. One was present at 30 cm while the other was present at 60 cm proximal to the ileocecal junction. A moderate amount of bilious collection was present in the peritoneal cavity. Several mucosal ulcers were present in the entire small intestine and the transverse colon as well; they presented as diffuse small whitish to yellowish oval, transverse flat lesions. A few of them had impending perforation. A biopsy was sent from the ulcer margin, mesenteric lymph node, and skin lesion on the forearm. Biopsy revealed a non-specific ileal ulcer with reactive lymphadenitis and stratified squamous epithelium with basal pigmentation in the epidermis with perineural and peri adnexal lymphocytic infiltrates in the dermis.

**Figure 3:**
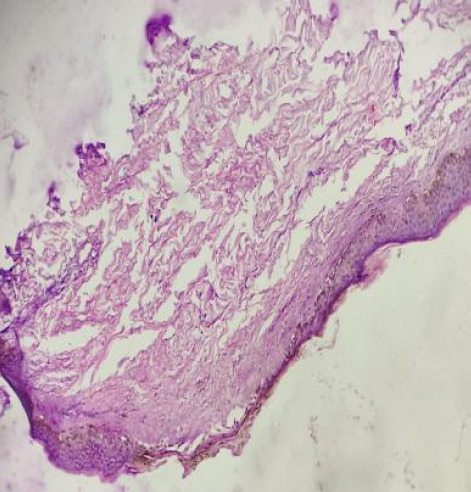
Atrophic focal area of the epidermis with sclerosis in the underlying dermis.

After one week of discharge, he was again brought to the emergency with a similar complaint. The X-ray abdomen revealed free gas under the diaphragm this time as well. USG showed multiple dilated bowel loops. A contrast-enhanced computed tomography (CECT) chest and abdomen were done, which confirmed edematous bowel loops in the right lower quadrant along with mild ascites and bilateral mild pleural effusion. Re-exploratory laparotomy and primary repair of ileal and jejunal perforations were performed. A skin biopsy was repeated, which revealed an atrophic focal area of the epidermis with sclerosis in the underlying dermis (Figure 3).

Despite the clinical findings and histopathology reports supporting the diagnosis of Degos disease, certain diseases need to be ruled out to conclude. Normal antinuclear antibodies ruled out systemic lupus erythematosus (SLE). The anti-saccharomyces antibody test, which indicates inflammatory bowel diseases, was also negative. A test for GeneXpert on an ascitic fluid sample produced negative findings. Given the above, the patient was diagnosed to have Degos disease and was started on low-dose aspirin.

The patient presented with pain in the abdomen again after two months and had to undergo exploratory laparotomy and repair of ileal and jejunal perforation. There were perforations of about 1 cm, one at 160 cm proximal to the ileocecal junction and the other at 140 cm proximal to the duodenojejunal flexure. There were several small ulcers all over the small intestine. During his fourth admission for acute pain in the abdomen, there was no significant finding on examination and initial investigations. However, the ultrasound abdomen revealed multiple prominent lymph nodes measuring up to 1.3x0.6 cm. White blood cells (WBC) count was lower and serum magnesium was higher than normal level. The patient developed septic shock and died on the fourth day of admission.

## DISCUSSION

MAP, also known as Degos disease or Kohlmeier-Degos disease, is a rare chronic thrombo-obliterative vasculopathy that primarily affects the skin, gastrointestinal tract, central nervous system, and occasionally other organs. The underlying cause of the disease is not well understood, and various theories have been proposed. However, the preferred mechanism appears to involve primary endothelial swelling with vascular thrombosis leading to tissue infarction.^4^

The disease typically presents with distinctive/ diagnostic skin lesions, starting as tiny solid papules that develop into porcelain-white depressed areas surrounded by a pink rim and fine blood vessels, which can coalesce and result in ulcerations.^7^ Our patient also had similar lesions as the initial presentation. The prognosis of MAP depends on the extent of systemic involvement, with the malignant form having a more unfavourable trajectory and affecting multiple organs.^1^ The benign cutaneous form can remain for years without affecting internal organs.^6^ However, in our case, intestinal perforation occurred just after a few months of the appearance of the skin lesions. Systemic involvement may occur simultaneously or later in the malignant form (for instance, multiple limited infarcts of the intestines, central nervous system, lungs, and eyes), which increases the risk of death by 50% within 2 to 3 years of the onset of symptoms.^2,3^ The bowel is affected in almost 50% of cases. In our case too, the patient displayed peritonitis symptoms in the emergency room, which prompted a very poor prognosis.

We considered the differential diagnoses namely SLE, tuberculosis, and inflammatory bowel disease, and ruled out the most common pathologies through a series of tests in our patient. As the pathophysiology of MAP remains unknown, there are no definitive treatment recommendations. However, antiplatelet medications, such as aspirin and dipyridamole, have shown some benefits in improving overall health and symptom management.^1,2^ In the case discussed, aspirin was the primary treatment modality used.

Overall, MAP is a rare and serious condition characterised by diagnostic skin lesions and potential multiorgan involvement. Further research is needed to better understand its underlying mechanisms and develop more effective treatments.
